# Risk factors for protein-caloric inadequacy in patients in an intensive care unit

**DOI:** 10.5935/0103-507X.20190067

**Published:** 2019

**Authors:** Celso Gustavo Ritter, Irla Maiara Silva Medeiros, Cláudia Sena de Pádua, Fernanda Raphael Escobar Gimenes, Patrícia Rezende do Prado

**Affiliations:** 1 Universidade Federal do Acre - Rio Branco (AC), Brasil.; 2 Secretaria do Estado de Saúde do Acre - Rio Branco (AC), Brasil.; 3 Departamento de Enfermagem Geral e Especializada, Escola de Enfermagem de Ribeirão Preto, Universidade de São Paulo - São Paulo (SP), Brasil.

**Keywords:** Nutrition therapy, Enteral nutrition, Nutrition, public health, Nutrition assessment, Risk factors, Critical illness, Intensive care units

## Abstract

**Objective:**

To evaluate the risk factors for protein-caloric inadequacy in critically ill patients.

**Methods:**

Prospective cohort study of patients hospitalized in an adult intensive care unit between February and November 2017. Patients were followed for 7 days. The conditional probability of inadequacy was calculated using the Kaplan-Meier method and the 95% log-rank test. To assess the risk of inadequacy, crude and adjusted hazard ratios (HR) were calculated using Cox regression with a 95% confidence interval.

**Results:**

Of the 130 patients, 63.8% were male, 73.8% were <60 years of age, and 49.2% were diagnosed with trauma. The mean APACHE II score was 24 points, and 70.0% of the patients had a protein-caloric adequacy >80%. In the univariate analysis, the significant variables for inadequacy were use of vasoactive drugs, interruptions of diet and failure to initiate nutrition early. In the final model, patients who presented with vomiting/gastric residue (adjusted HR = 22.5; 95%CI 5.14 - 98.87) and fasting for extubation (adjusted HR = 14.75; 95%CI 3.59 - 60.63) and for examinations and interventions (adjusted HR = 12.46; 95%CI 4.52 - 34.36) had a higher risk of not achieving protein-caloric adequacy.

**Conclusion:**

Achievement of nutritional goals > 80.0% occurred in 70.0% of patients. The risk factors for protein-caloric inadequacy were nutritional interruptions, especially due to vomiting/gastric residue and fasting for extubation, exams and surgical procedures.

## INTRODUCTION

Protein supply has been promoted in several studies as a fundamental element of recovery in critically ill patients. The stress state, the possible presence of sepsis, organ dysfunction and other complications inherent to patients in the intensive care unit (ICU) trigger multisystemic disorders and changes in macronutrient metabolism as an endocrine-metabolic response. Thus, in critically ill patients, there is an increase in the expenditure of energy and the use of body reserves, especially skeletal muscle protein, which may result in increased protein-caloric demand and imbalance.^(1,2)^

Therefore, nutritional inadequacy, together with other factors such as immunosuppression, weakness and poor healing, contribute to reduced patient survival, increased length of stay in the ICU and higher hospital costs. Protein-caloric adequacy is essential in the acute phase of disease to preserve or reduce the loss of lean body mass, a factor that impacts patient prognosis.^(3,4)^

In this sense, nutritional therapy (NT) is the main tool for ensuring the adequate protein-caloric intake of critically ill patients by calculating their nutritional needs and dietary allowances. However, adequacy can be hindered by several conditions that affect nutritional supply, such as the patient's clinical condition and the performance of therapeutic procedures in the ICU; such factors must be identified to ensure protein-caloric adequacy and improved patient survival.^(5-8)^

Enteral nutrition (EN) aims to ensure greater accuracy and safety of NT through the infusion of nutrients, such as proteins and calories. Individuals who require a higher nutritional intake are often unable to ingest or digest food orally, as in the case of patients under sedation or mechanical ventilation and those with some types of trauma.^(6,9)^ Thus, the following question has arisen: Is the protein-caloric intake of critically ill patients in the ICU adequate? If it is not adequate, what are the risk factors for protein-caloric inadequacy in critically ill patients?

The objective of this study was to evaluate the risk factors for protein-caloric inadequacy in critically ill patients receiving NT in the ICU.

## METHODS

This prospective cohort study included patients admitted to the adult ICU of a general urgency and emergency hospital in the city of Rio Branco, Acre, in the Brazilian Western Amazon. Data were collected between February and November 2017.

Individuals who had received exclusive EN for at least 72 hours (3 days) were included. Patients who did not have an arterial blood gas analysis on admission or within 24 hours after admission to the ICU were also excluded due to the inability to calculate their Acute Physiology and Chronic Health Evaluation II (APACHE II) score.

Twelve clinical criteria are used to calculate the APACHE II score: temperature, mean blood pressure, heart rate, respiratory rate, oxygenation (fraction of inspired oxygen (FiO_2_) and partial pressure of oxygen (PO_2_), arterial pH, serum sodium (Na^+^), serum potassium (K^+^), serum creatinine with or without acute renal failure, hematocrit, leukocytes, Glasgow coma scale, age and comorbidities). A value of zero to 6 points was attributed to each clinical criteria; the scores for all criteria were summed and used to classify the patient into one of eight severity levels for risk of death, with intervals ranging from 4% to 85% based on data from the first 24 hours after ICU admission.^(10)^

The data were collected using an instrument previously developed by the resident nutritionist with dependent and independent variables that assessed protein-caloric adequacy over 7 days of follow-up. Data were collected daily for all patients who met the inclusion criteria from the medical records at admission, the nursing care systematization tool and the unit's nutritional progression protocol.

Protein-caloric adequacy was considered as the dependent variable, and the following independent variables were analyzed: age, sex, diagnosis (clinical or trauma), APACHE II score, daily total energy value (TEV), amount of protein, use of vasoactive drugs, gastrointestinal tract complications, interruption of diet (vomiting/gastric residue, fasting for exams and medical procedures/orotracheal extubation), hemodialysis, early nutrition (NT started between 24 and 48 hours after ICU admission) and clinical outcome (discharge or death).^(11)^

Adequate caloric intake consisted of between 25 and 30kcal per kg of estimated weight, reaching 40kcal/kg/day in large burn patients and 22 to 25kcal/kg/day in obese patients using the ideal weight.^(2)^ The pocket formula was used to determine the energy needs of each patient, as recommended by the Brazilian Society of Parenteral and Enteral Nutrition (Sociedade Brasileira de Nutrição Parenteral e Enteral (SBNPE)). Regarding the enteral dietary volume, progressive infusion started at 500mL on the first day of enteral NT (ENT) and was increased to 1,500mL over 72 hours using an open or closed system. The protein content was considered adequate at between 1.2 and 2.0g/kg/day, depending on the patient's metabolic status, and 1.5g/kg/day was established as a standard.^(2,12)^ Protein-caloric intake was considered adequate when it was > 80% of the nutritional prescription, as recommended in the guidelines of the American Society for Parenteral and Enteral Nutrition (ASPEN).

The study sample size was calculated using the following formula for a finite population and considering a 95% confidence interval (95%CI), 5% random error, and 70.8% prevalence of adequacy, according to a study conducted at an ICU in the city of Rio Branco,^(13)^ and a population of 200 patients, according to the mean number of intubated patients receiving EN who were hospitalized in that unit over a 1-year period. Thus, the calculated sample size was 123 patients.^(14)^ Data on 130 patients were collected to account for the probability of loss.

$$n=\frac{N\cdot Z^{2}\cdot p\cdot \left(1-p\right)}{\left(N-1\right)\cdot e^{2}+Z^{2}\cdot p\cdot \left(1-p\right)}$$

Where n corresponds to the calculated sample (123), N represents the population (200 intubated patients per year), Z represents the normal variable standardized to the confidence level (95%), p indicates the prevalence (70.8%),^(13)^ and e indicates the sampling error (5%).

The data were organized in Excel 2010 (Microsoft, USA) and analyzed using the Statistical Package for the Social Sciences (SPSS), version 22.0 (SPSS Corp., Chicago, IL, USA).

Time zero (T0) for this cohort was the first day of exclusive ENT. The follow-up time (ΔT) was 7 days. Data analysis was performed using the Kaplan-Meier method to estimate the conditional probability of protein-caloric inadequacy, using the 95% log-rank test to evaluate the differences between the curves.

Cox regression analysis was used to assess the risk factors associated with protein-caloric inadequacy, yielding crude and adjusted risks and their respective 95%CIs.

The final model was designed to assess risk factors for protein-caloric inadequacy in critically ill ICU patients. The independent variables that demonstrated statistical significance in the univariate analysis were included in the Cox multivariate regression model, with a p-value < 5% as the criterion for inclusion in and a p-value > 10% as the criterion for exclusion from the model.

This project was approved by the Research Ethics Committee of the *Fundação Hospital Estadual do Acre* (FUNDHACRE) under CAAE 47577215.2.0000.5009.

## RESULTS

Of the 130 patients, 63.8% were male, 73.8% were younger than 60 years, 49.2% had a trauma diagnosis, and 48.5% had APACHE II probability of death above 40%. All patients were under mechanical ventilation on the first day of follow-up, 79.2% used vasoactive drugs, and 12.3% underwent hemodialysis at any time during the evaluation period. The mean length of hospital stay was 19.3 ± 15.4 days, and 24.6% of the patients died. Regarding nutritional demands, 70.0% of the patients achieved protein-caloric adequacy (> 80% of the nutritional goals), and 84.6% received early nutrition (Table 1).

**Table 1:** Epidemiological and clinical characteristics of patients in the intensive care unit (n = 130)

Variable	n (%)
Age (years)	
< 60	96 (73.8)
≥ 60	34 (26.2)
Sex	
Male	83 (63.8)
Female	47 (36.2)
Diagnosis	
Clinical	66 (50.8)
Trauma	64 (49.2)
APACHE II (points)	
< 40% (0 - 24)	67 (51.5)
≥ 40% (25 - 100)	63 (48.5)
Vasoactive drugs	
Yes	103 (79.2)
No	27 (20.8)
Hemodialysis	
Yes	16 (12.3)
No	114 (87.7)
TEV (kcal)/kg/day	
< 2,000	23 (17.7)
≥ 2,000	107 (82.3)
Protein g/kg/day	
< 1.5	14 (10.8)
≥ 1.5	116 (89.2)
Early nutrition < 48 hours	
Yes	110 (84.6)
No	20 (15.4)
Adequacy at 72 hours	
< 80%	39 (30.0)
≥ 80%	91 (70.0)
Diet interruption at 72 hours	
No interruption	90 (69.2)
Vomiting/gastric residue	4 (3.1)
Surgery/TCT	6 (4.6)
Extubation	6 (4.6)
Other/exams	24 (18.5)
Outcome	
Discharge	98 (75.4)
Death	32 (24.6)

APACHE - Acute Physiology and Chronic Health Evaluation II; TEV - total energy value; TCT - tracheostomy.

The highest probability of protein-caloric inadequacy was observed for the most severe patients who used vasoactive drugs (3.7% *versus* 3.1% in patients not using these drugs, log-rank = 0.01), for patients with an APACHE II score indicating a > 40% probability of death and for those who underwent hemodialysis, although the latter two factors did not present statistical significance. The same finding was observed for patients who experienced interruption of the diet, especially when they exhibited vomiting/gastric residue or underwent fasting for endotracheal extubation, surgery and exams (33.3%, 25.0%, 16.7% and 12.5% probability of failure, respectively) (log-rank < 0.0001), and for those who did not receive early initiation of nutrition (5.0% *versus* 3.8% compared to patients who received nutrition between 24 and 48 hours after ICU admission; log-rank = 0.01) (Table 2).

**Table 2:** Conditional probability of protein-caloric inadequacy in intensive care unit patients

Variables	Probability of protein-caloric inadequacy on Day 7 (%)	Log-rank (95%CI)
Age (years)		
< 60	3.2	0.59
≥ 60	3.0	
Sex		
Male	4.9	0.54
Female	6.8	
Diagnosis		
Clinical	4.7	0.91
Trauma	1.6	
APACHE II (points)		
< 40% (0 - 24)	4.6	0.57
≥ 40% (25 - 100)	5.0	
Vasoactive drugs		
Yes	3.7	0.01
Not applicable	3.1	
Hemodialysis		
Yes	6.2	0.15
Not applicable	2.7	
TEV (kcal)/kg/day		
< 2,000	3.5	0.49
≥ 2,000	2.9	
Protein (g)/kg/day		
< 1.5	2.8	0.87
≥ 1.5	10.0	
Early nutrition < 48 hours		
Yes	3.8	0.01
Not applicable	5.0	
Adequacy at 72 hours		
< 80%	-	-
≥ 80%	-	
Diet interruption at 72 hours		
No interruption	1.1	< 0.0001
Vomiting/gastric residue	33.3	
Surgery/TCT	16.7	
Extubation	25.0	
Other/exams	12.5	
Outcome		
Discharge	2.1	0.61
Death	6.7	

95%CI - 95% confidence interval; APACHE - Acute Physiology and Chronic Health Evaluation II; TEV - total energy value; TCT - tracheostomy.

In the final model, the risk factors for protein-caloric inadequacy were interruptions in the diet, especially due to vomiting/gastric residue (adjusted HR = 22.5; 95%CI 5.14 - 98.87), fasting for extubation (adjusted HR = 14.75; 95%CI 3.59 - 60.63), fasting for exams and interventions (adjusted HR = 12.46; 95%CI 4.52 - 34.36) and elective surgical procedures (adjusted HR = 11.9; 95%CI 3.60 - 39.71) (Table 3).

**Table 3:** Crude and adjusted hazard ratios for risk of inadequate nutrition therapy

Variables	Crude HR (IC95%)	Adjusted HR (IC95%)
Vasoactive drugs		
Yes	2,53 (1,28 - 4,99)	1,14 (0,53 - 2,46)
Diet interruption at 72 hours		
No interruption	1	1
Vomiting/gastric residue	23.05 (5.39 - 98.62)	22.5 (5.14 - 98.87)
Surgery/TQT	12.11 (3.65 - 40.09)	11.9 (3.60 - 39.71)
Extubation	16.08 (4.23 - 61.14)	14.75 (3.59 - 60.63)
Other/exams	12.88 (4.83 - 34.34)	12.46 (4.52 - 34.36)
Early nutrition at < 48 hours	1	1
No	1.26 (0.60 - 2.62)	0.98 (0.44 - 2.15)

HR - hazard ratio; TQT - tracheostomy.

## DISCUSSION

Similar to the profile of patients in this ICU study, a retrospective multicenter study conducted by the Clinical Evaluation Unit in Ontario, Canada, involving 17,154 individuals from 923 ICUs, also identified a predominance of male patients in Canadian ICUs as well as a mean age of 60 years and mean APACHE II score of 22 points; however, the majority of the patients in that study had a clinical diagnosis (63%), and 19.0% died within 60 days of hospitalization.^(15)^

In Brazil, the epidemiological profile of the present study resembled that reported in an observational study of 212 patients in a university hospital in Belém, Pará, in which male sex was also predominant, the mean age was 49.2 years, and trauma was responsible for only 0.5% of the hospital admissions. Another study with 201 patients published by the Multidisciplinary Nutritional Therapy Team of Sírio Libanês Hospital in the city of São Paulo in 2014 revealed a mean age of 75.4 years and a predominance of clinical diagnoses.^(7,16)^ The patient profile observed in this study can be explained by the fact that the study hospital is a reference for urgent care and emergencies throughout the state and has a higher incidence of admission of young, male victims of traffic accidents and other traumatic events.^(17)^

Patient severity also did not correlate with inadequate protein and energy supply in a prospective observational study conducted in Amsterdam, Netherlands, where 60.0% of the patients were victims of trauma, and the mean APACHE II score was 23 points.^(18)^ The results of this study were similar to those published by the Multi-professional Health Residency Team of the Federal University of Goiás, in which 52.6% of the sample had an APACHE II score of 15-23 points, and to data from the Multi-Professional Residency Group of the Federal University of Rio Grande do Sul, which reported a mean APACHE II score of 24.1 ± 9.6 points.^(19,20)^ However, it is noteworthy that in our study, patients with an APACHE II score indicating a > 40% likelihood of mortality had a higher probability of inadequate NT, but the association was not statistically significant.

Still regarding patient severity, the use of vasoactive drugs had no relationship with the risk of protein-caloric inadequacy in this study or in a retrospective cohort study conducted in the medical-surgical ICU of a university hospital in Peta Kivah, Israel, where 39.0% of the patients used vasopressors.^(21)^ This suggests that although hemodynamic instability contraindicates the start of EN, it is not a risk factor for nutritional inadequacy. There was also no difference in the start of nutrition based on whether the cause of hospitalization was clinical or traumatic; although the patients with clinical diagnosis had a higher conditional probability of failure, the difference was not statistically significant.

Mortality in the ICU may vary due to factors such as the hospitalized patient's profile and severity, in addition to the objective of the study and the population analyzed. The mortality rate found in this study was low, as a large proportion of recent studies have reported mortality rates above 40.0%.^(16,20,21)^ However, it should be considered that the patient profile of the unit and the fact that trauma was the predominant cause of hospitalization may favor recovery and discharge from the ICU when compared to older populations and/or those with chronic clinical diagnoses.

Regarding protein-caloric adequacy, a study conducted at the University Hospital of the University of São Paulo reported 100% achievement of nutritional goals for 80.0% of the sample; this is in contrast to the results of the present study, in which 70.0% of patients had achieved their nutritional goals at 72 hours after the start of NT, and is also higher than the results reported in a study performed at Hospital das Clínicas of Porto Alegre, where only 50.0% of the population met their nutritional goals.^(6,20)^ In turn, a study conducted at a general hospital in Taichung, Taiwan, found that patients met 65.0% of the prescribed energy goals - approximately 934kcal/day. Based on this rate, the authors concluded that there were decreases in mortality at days 14 and 28 of the hospital stay when the energy supply was ≥ 800kcal/day, and the same may be suggested by our study.^(22)^

Regarding early nutrition, our results were higher than those reported by Hospital das Clínicas of Porto Alegre, where 63.0% of patients received early nutrition; that study's finding was lower than that of the Multiprofessional Health Team of Fluminense Federal University, where the 74 individuals in the sample started nutrition early, within a mean of 28.8 ± 38.6 hours.^(20,23)^ In Amsterdam, 16.0 hours was the mean time before the start of NT, which may have favored the finding that 95.0% of the sample achieved their protein goals within 4 days of follow-up.^(18)^ Thus, early nutrition is essential for ICU patients to decrease the probability of protein-caloric inadequacy, as shown in this study.^(2,11)^

The main cause of the inadequate attainment of nutritional goals observed in this ICU was the interruption of the diet, especially due to gastrointestinal symptoms such as vomiting and gastric residue, which led to a 22.5-fold higher risk of protein-caloric inadequacy. Similar data were identified in a study published by the Federal University of Goiás and one conducted in Ontario, which reported that fasting for procedures was the main reason for diet interruption. Similar to these findings, several other studies reported gastric residue as one of the most prevalent complications.^(2,19,24)^ Thus, the results show that the scheduling of therapeutic procedures and attention to preventable causes of nutritional discontinuation are challenges in ICUs in several countries.^(15,23)^

In this cohort, after gastrointestinal manifestations, patients who fasted for extubation and underwent exams/procedures had a higher risk of inadequacy (14.75 and 12.46 times, respectively). The demands of the ICU care routine deserve attention because feeding must be suspended before and after such procedures and because procedures may not occur on the scheduled date due to costs, patient severity, logistics, technological issues and the availability of human and material resources. The development and application of a standard protocol established by each unit to monitor the duration of diet suspension, as has occurred in other ICUs, could provide a strategy for controlling these disruptions and thus reducing the risk of protein-caloric inadequacy.^(7,19,25)^

In terms of limiting factors of this study, the patients' nutritional statuses were not screened or assessed, nor was the number of hours of diet suspension calculated. The factors that could be investigated were limited to the reasons for diet suspension. Another factor that limited the achievement of nutritional goals, especially protein goals, was the unavailability or discontinuation of nutritional formulas for certain periods, which made achieving individually prescribed and calculated goals challenging.

In turn, the awareness of the multidisciplinary ICU staff where the study was conducted is a favorable point. The professionals are sensitive to and have interest in resuming NT and monitoring identified risk factors. The use of the evaluation protocol and the nutritional progression previously developed for the profile of patients treated at this ICU also reinforce the importance of NT, showing respect for individual needs and for supervising the dietary supply. In addition, the present study used a significant sample of critically ill patients and important data that identified the causes of protein-caloric inadequacy, bringing attention to factors that required intervention and monitoring by the multidisciplinary team.

## CONCLUSION

Protein-caloric adequacy > 80% was achieved by the majority of patients, with decreasing enteral nutrition between days 3 and 7 of follow-up. Diet interruptions were the main risk factors for protein-caloric inadequacy and were associated with gastrointestinal disruptions and fasting for procedures, interventions and tests.

An understanding of the factors that limit the scope of nutritional therapy is important for formulating strategies to improve supply conditions, decrease the protein and calorie deficit in these patients and, consequently, reduce the risk of hospital malnutrition. These findings may contribute to the development and adaptation of instruments for use by multidisciplinary teams to monitor risk factors, thus increasing the adequacy of nutritional therapy and recognizing the role of nutrition in the recovery of critically ill patients.
